# Serum resistin as an independent marker of aortic stiffness in patients with coronary artery disease

**DOI:** 10.1371/journal.pone.0183123

**Published:** 2017-08-14

**Authors:** Ji-Hung Wang, Chung-Jen Lee, Chiu-Fen Yang, Yu-Chih Chen, Bang-Gee Hsu

**Affiliations:** 1 Division of Cardiology, Buddhist Tzu Chi General Hospital, Hualien, Taiwan; 2 School of Medicine, Tzu Chi University, Hualien, Taiwan; 3 Department of Nursing, Tzu Chi University of Science and Technology, Hualien, Taiwan; 4 Division of Nephrology, Buddhist Tzu Chi General Hospital, Hualien, Taiwan; Institute for Nutritional Sciences, CHINA

## Abstract

**Background:**

Subjects with higher carotid–femoral pulse wave velocity (cfPWV) will be at an increased risk for cardiovascular (CV) events in future. Resistin is an inflammatory mediator and a biomarker of CV diseases. We evaluated the association between serum resistin and aortic stiffness in patients with coronary artery disease (CAD).

**Methods:**

A total of 104 patients with CAD were enrolled in this study. cfPWV was measured using the SphygmoCor system. Patients with cfPWV >10 m/s were defined as the high aortic stiffness group.

**Results:**

Thirty-seven patients (35.6%) had high aortic stiffness and higher percentages of diabetes (*p* = 0.001), were of older age (*p* = 0.001) and had higher waist circumference (*p* < 0.001), systolic blood pressure (*p* = 0.027), pulse pressure (*p* = 0.013), high-sensitivity C-reactive protein (*p* < 0.001) and resistin levels (*p* < 0.001) but lower estimated glomerular filtration rate (*p* = 0.009) compared to subjects with low aortic stiffness. After adjusting for factors significantly associated with aortic stiffness by multivariate logistic regression analysis, serum resistin (odds ratio = 1.275, 95% confidence interval: 1.065–1.527, *p* = 0.008) was also found to be an independent predictor of aortic stiffness in patients with CAD.

**Conclusions:**

Serum resistin level is a biomarker for aortic stiffness in patients with CAD.

## Introduction

Arterial stiffening due to loss of arterial compliance results in a more rapid travel time and hence a higher pulse wave velocity [1]. A majority of epidemiological studies have demonstrated the predictive value of aortic stiffness as an independent predictor of cardiovascular (CV) morbidity and mortality by measuring the carotid–femoral pulse wave velocity (cfPWV), independent of traditional risk factors [2–4]. cfPWV was included in the 2013 European Society of Hypertension and the European Society of Cardiology (ESH/ESC) guidelines for the management of hypertension, and cfPWV values of >10 m/s were described as influencing CV prognosis, besides blood pressure (BP) [5], and also as a useful vascular biomarker for primary and secondary CV disease prevention (Class IIa; Level of Evidence A) by the Association for Research into Arterial Structure and Physiology (ARTERY) Society [1].

Resistin named for its ability to resist insulin action has an important link between obesity, insulin resistance and diabetes [6, 7]. Moreover, additional reports suggest that resistin has a pathogenic role in the development and progression of atherosclerosis and coronary artery disease (CAD) [6–8]. Recently, a study from South Africa reported that plasma resistin is independently associated with cfPWV in a community-based sample of participants having never been treated with antihypertensive therapy and with a high prevalence of obesity [9]. Our previous study also noted serum leptin level could be a predictor for aortic stiffness in hypertensive patients [10]. Although aortic stiffness has been known to predict CV morbidity and mortality and resistin is associated with CAD, the relationship between aortic stiffness and serum resistin in patients with CAD is not much clear. Therefore, we sought to examine the relationship between serum resistin levels and aortic stiffness in patients with CAD.

## Materials and methods

### Patients

A total of 104 patients with CAD were enrolled from a medical centre in Hualien, Taiwan, from January through December 2012 in this study. CAD was defined as >50% stenosis in any segment by coronary angiography by medical record. BP was measured for all participants by trained staff in the morning using standard mercury sphygmomanometers with appropriate cuff sizes after making the patients rest for at least 10 minutes. Systolic BP (SBP) and diastolic BP (DBP) were measured three times at 5-min intervals and were averaged for analysis. Pulse pressure was calculated by subtracting DBP from SBP. Hypertension was diagnosed as SBP ≥ 140 mmHg and/or DBP ≥ 90 mmHg or having received any anti-hypertensive drugs in the past 2 weeks. Patients were diagnosed with diabetes mellitus (DM) if their fasting plasma glucose was either ≥126 mg/dl or if they were using oral hypoglycaemic medications or insulin [11]. The Protection of the Human Subjects Institutional Review Board of Tzu-Chi University and Hospital approved this study (IRB-099-97). All participants provided their informed consents before participating in this study. Study participants were recruited in the cardiovascular outpatient department and participants were included if they had CAD history. Participants were excluded if they had an acute infection, acute myocardial infarction, pulmonary oedema at the time of blood sampling or were taking calcium, active vitamin D metabolites, bisphosphonates, teriparatide or estrogens or if they declined to provide informed consent for the study.

### Anthropometric analysis

Body weight of the participants was measured with light clothing and without shoes to the nearest 0.5 kg, and body height was measured to the nearest 0.5 cm. Waist circumference was measured using a tape around the waist from the point between the lowest rib and the hip bone with the hands on the hips. Body mass index (BMI) was calculated as the weight in kilograms divided by the height in square metres [10, 12–15].

### Biochemical investigations

Approximately 5 ml of blood samples collected from each participant after an overnight fasting was immediately centrifuged at 3000 *g* for 10 min. Serum levels of blood urea nitrogen (BUN), creatinine (Cre), fasting glucose, total cholesterol (TCH), triglycerides (TG), high-density lipoprotein cholesterol (HDL-C), low-density lipoprotein cholesterol (LDL-C) and high-sensitivity C-reactive protein (hs-CRP) were measured using an auto-analyser (COBAS Integra 800, Roche Diagnostics, Basel, Switzerland) [10, 12–15]. Concentrations of human serum resistin were determined using a commercially available enzyme immunoassay (EIA) kit (SPI-BIO, Montigny le Bretonneux, France) [16]. The estimated glomerular filtration rate (eGFR) was calculated by the Chronic Kidney Disease Epidemiology Collaboration (CKD-EPI) equation.

### Aortic stiffness by carotid-femoral pulse wave velocity measurements

Measurements of cfPWV for assessing the aortic stiffness were performed using pressure applanation tonometry (SphygmoCor system, AtCor Medical, Australia) as previously described [10, 12–15]. These measurements were performed for all participants in the morning in the supine position after a minimum 10-min rest in a quiet and temperature-controlled room. Recordings were made simultaneously with an ECG signal, which provided an *R*-timing reference. Pulse wave recordings were performed consecutively at two superficial artery sites (carotid–femoral segment). The carotid–femoral distance was obtained by subtracting the distance from the carotid measurement site to the sternal notch from the distance from the sternal notch to the femoral measurement site. Integral software was used for processing each set of pulse wave and ECG data to calculate the mean time difference between *R*-wave and pulse wave on a beat-to-beat basis, with an average of 10 consecutive cardiac cycles. The cfPWV was calculated using the distance and mean time difference between the two recorded points. Quality indices, included in the software, were set to ensure uniformity of data. In this study, cfPWV values of >10 m/s were used to define the group with high aortic stiffness [5, 10, 17].

### Statistical analysis

Data were tested for normal distribution using the Kolmogorov–Smirnov test. Normally distributed data are expressed as mean ± standard deviation (SD), and comparisons between patients were performed using the Student’s independent *t*-test (two-tailed). Data that were not normally distributed are expressed as median and interquartile ranges, and comparisons between patients were performed using the Mann–Whitney U test (TG, fasting glucose, BUN, Cre and hs-CRP). Data expressing the number of patients were analysed by the χ^2^ test. Because TG, fasting glucose, BUN, Cre and hs-CRP were not normally distributed, they underwent base 10 logarithmic transformations to achieve normality. Clinical variables that correlated with serum resistin levels in patients with CAD were evaluated using univariate linear regression analysis. Variables that were significantly associated with resistin levels in patients with CAD were tested for independency by the multivariate forward stepwise regression analysis. Variables that were significantly associated with aortic stiffness were tested for independency by the multivariate logistic regression analysis (adapted factors were DM, age, waist circumference, SBP, pulse pressure, GFR, hs-CRP and resistin). Data were analysed using SPSS for Windows (version 19.0; SPSS Inc., Chicago, IL, USA). A *p* value < 0.05 was considered as statistically significant.

## Results

Clinical characteristics of the 104 patients with CAD are shown in Table 1. A total of 50 patients (48.1%) had DM and 82 patients (78.8%) had hypertension. Thirty-seven patients (35.6%) were defined as having high aortic stiffness. Patients in the high aortic stiffness group had a higher percentage of DM (p = 0.001) compared to the low aortic stiffness group. Patients with CAD with high aortic stiffness were older (p = 0.001) and had higher waist circumference (p < 0.001), SBP (p = 0.027), pulse pressure (p = 0.013), hs-CRP (p <0.001) and serum resistin levels (p < 0.001) but had lower eGFR (p = 0.009) compared to those with low aortic stiffness. The drugs used by the patients included angiotensin receptor blockers (ARB; n = 44; 42.3%), angiotensin-converting enzyme inhibitors (ACEi; n = 30; 28.8%), calcium channel blockers (CCB; n = 31; 29.8%), β-blockers (n = 61; 58.7%), statins (n = 72; 69.2%) and fibrate (n = 18; 17.3%). There was no statistically significant difference in sex and use of ACEi, ARB, β-blockers, CCB, statins or fibrate between the two groups.

**Table 1:** Clinical variables of the 104 patients with coronary artery disease with high or low aortic stiffness.

Characteristics	All Patients (*n* = 104)	Low Aoritc Stiffness Group (*n* = 67)	High Aortic Stiffness Group (*n* = 37)	p value
Age (years)	65.63 ± 9.17	63.39 ± 8.96	69.682 ± 8.19	0.001*
Height (cm)	161.04 ± 8.23	161.61 ± 7.50	160.00 ± 9.43	0.341
Body weight (kg)	67.77 ± 11.84	67.75 ± 12.48	67.80 ± 10.75	0.982
Waist circumference (cm)	92.56 ± 9.27	90.15 ± 8.70	96.92 ± 8.75	< 0.001*
Body mass index (kg/m^2^)	26.03 ± 3.40	25.79 ± 3.40	26.47 ± 3.40	0.339
cfPWV (m/s)	9.40 ± 2.72	7.82 ± 1.33	12.25 ± 2.20	< 0.001*
SBP (mmHg)	131.82 ± 18.54	128.85 ± 16.26	137.19 ± 21.27	0.027*
DBP (mmHg)	72.40 ± 10.24	72.43 ± 10.32	72.35 ± 10.23	0.969
Pulse pressure (mmHg)	59.41 ± 16.67	56.42 ± 14.72	64.84 ± 18.73	0.013*
Total cholesterol (mg/dl)	166.01 ± 35.97	170.25 ± 37.73	158.32 ± 31.59	0.106
Triglyceride (mg/dl)	118.00 (89.25–162.00)	119.00 (90.00–190.00)	117.00 (89.00–152.00)	0.337
HDL-C(mg/dl)	45.21 ± 12.24	46.37 ± 12.15	43.11 ± 12.28	0.194
LDL-C(mg/dl)	95.82 ± 26.69	97.52 ± 26.64	92.73 ± 26.87	0.383
Fasting glucose (mg/dl)	111.00 (96.25–145.25)	108.00 (97.00–134.00)	114.00 (95.50–163.00)	0.541
Blood urea nitrogen (mg/dl)	16.00 (13.00–19.00)	15.00 (13.00–18.00)	16.00 (13.00–21.50)	0.150
Creatinine (mg/dl)	1.10 (0.90–1.30)	1.00 (0.90–1.20)	1.20 (0.90–1.40)	0.118
eGFR (ml/min)	68.44 ± 19.50	72.10 ± 17.27	61.81 ± 21.69	0.009*
hs-CRP (mg/dl)	0.19 (0.14–0.26)	0.17 (0.13–0.21)	0.25 (0.20–0.36)	< 0.001*
Resistin (ng/ml)	7.50 ± 3.99	6.07 ± 2.85	10.09 ± 4.46	< 0.001*
Male, n (%)	78 (75.0%)	50 (74.6%)	28 (75.7%)	0.906
Diabetes, n (%)	50 (48.1%)	24 (35.8%)	26 (70.3%)	0.001*
Hypertension, n (%)	82 (78.8%)	50 (74.6%)	32 (86.5%)	0.156
ACE inhibitor use, n (%)	30 (28.8%)	18 (26.9%)	12 (32.4%)	0.549
ARB use, n (%)	44 (42.3%)	26 (38.8%)	18 (43.6%)	0.331
β-blocker use, n (%)	61 (58.7%)	38 (56.7%)	23 (62.2%)	0.589
CCB use, n (%)	31 (29.8%)	17 (25.4%)	14 (37.8%)	0.183
Statin use, n (%)	72 (69.2%)	49 (73.1%)	23 (63.2%)	0.246
Fibrate use, n (%)	18 (17.3%)	9 (13.4%)	9 (24.3%)	0.160

Values for continuous variables are given as mean ± standard deviation and tested by Student’s t-test; variables not normally distributed are given as median and interquartile range and tested by Mann–Whitney U test; values are presented as number (%) and analysis was done using the chi-square test.

AS, arterial stiffness; cfPWV, carotid–femoral pulse wave velocity; SBP, systolic blood pressure; DBP, diastolic blood pressure; HDL-C, high-density lipoprotein cholesterol; LDL-C, low-density lipoprotein cholesterol; eGFR, estimated glomerular filtration rate; hs-CRP, high-sensitivity C-reactive protein; ACE, angiotensin-converting enzyme; ARB, angiotensin receptor blocker; CCB, calcium channel blocker.

*p < 0.05 was considered statistically significant.

Patients with CAD and DM had higher serum resistin levels (p = 0.003) than those without DM comorbidity. There was no statistically significant difference in serum resistin levels in terms of sex, hypertension and use of ACEi, ARB, β-blockers, CCB, statins or fibrate (Table 2).

**Table 2:** Clinical characteristics and serum resistin levels of the 104 patients with coronary artery disease.

Characteristic		Number (%)	Resistin (ng/ml)	p value
Sex	Male	78	7.57 ± 4.21	0.755
	Female	26	7.29 ± 3.30	
Diabetes	No	54	6.39 ± 3.03	0.003*
	Yes	50	8.69 ± 4.56	
Hypertension	No	22	8.94 ± 4.20	0.057
	Yes	82	7.11 ± 3.87	
ACE inhibitor	No	74	7.59 ± 4.09	0.702
	Yes	30	7.26 ± 3.80	
ARB	No	60	7.63 ± 3.97	0.690
	Yes	44	7.32 ± 4.06	
β-blocker	No	43	7.27 ± 3.98	0.622
	Yes	61	7.66 ± 4.02	
CCB	No	73	7.57 ± 3.95	0.793
	Yes	31	7.34 ± 4.15	
Statin	No	32	6.92 ± 3.64	0.328
	Yes	72	7.76 ± 4.14	
Fibrate	No	86	7.46 ± 4.16	0.840
	Yes	18	7.67 ± 3.18	

Data are expressed as mean ± standard deviation and tested by Student’s t-test.

ACE, angiotensin-converting enzyme; ARB, angiotensin receptor blocker; CCB, calcium channel blocker.

* p < 0.05 was considered statistically significant after Student’s t-test.

Results of the univariate and multivariate linear analyses of clinical variables associated with serum resistin levels in patients with CAD are presented in Table 3. Age (r = 0.246; p = 0.012), waist circumference (r = 0.415; p < 0.001), logarithmically transformed Cre (log-Cre, r = 0.227; p = 0.020) and log-hs-CRP (r = 0.269; p = 0.006) positively correlated while eGFR (r = −0.251; p = 0.010) negatively correlated with serum resistin levels in patients with CAD. Multivariate forward stepwise linear regression analysis of the variables significantly associated with fasting serum resistin levels revealed that DM (β = 0.239; p = 0.006), age (β = 0.203; p = 0.019) and waist circumference (β = 0.384; p < 0.001) were independent predictors of resistin values in patients with CAD.

**Table 3:** Correlation between serum resistin levels and clinical variables among the 104 patients with coronary artery disease.

Variables	Resistin (ng/ml)			
	Univariate analysis		Multivariate analysis	
	*r*	p	Beta	p
Age (years)	0.246	0.012*	0.203	0.019*
Height (cm)	-0.134	0.174	-	-
Body weight (kg)	-0.117	0.238	-	-
Waist circumference (cm)	0.415	< 0.001*	0.384	< 0.001*
Body mass index (kg/m^2^)	-0.042	0.669	-	-
SBP (mmHg)	0.039	0.693	-	-
DBP (mmHg)	0.010	0.919	-	-
Pulse pressure (mmHg)	0.037	0.706	-	-
Total cholesterol (mg/dl)	-0.109	0.272	-	-
Log-Triglyceride (mg/dl)	-0.089	0.367	-	-
HDL-C(mg/dl)	0.001	0.989	-	-
LDL-C(mg/dl)	-0.066	0.506	-	-
Log-glucose (mg/dl)	0.004	0.969	-	-
Log-Blood urea nitrogen (mg/dl)	0.099	0.318	-	-
Log-Creatinine (mg/dl)	0.227	0.020*	-	-
eGFR (ml/min)	-0.251	0.010*	-	-
Log-hs-CRP (mg/dl)	0.269	0.006*	-	-

Data of triglyceride, glucose, BUN, Cre and hs-CRP levels showed skewed distribution and therefore were log-transformed before analysis.

Analysis of data was done using the univariate linear regression analyses or multivariate stepwise linear regression analysis (adapted factors were diabetes, age, waist circumference, log-creatinine, GFR and hs-CRP).

cfPWV, carotid–femoral pulse wave velocity; SBP, systolic blood pressure; DBP, diastolic blood pressure; HDL-C, high-density lipoprotein cholesterol; LDL-C, low-density lipoprotein cholesterol; eGFR, estimated glomerular filtration rate; hs-CRP, high-sensitivity C-reactive protein.

*p < 0.05 was considered statistically significant.

Moreover, after adjusting for factors significantly associated with aortic stiffness (DM, age, waist circumference, SBP, pulse pressure, eGFR, hs-CRP and resistin) by multivariate logistic regression analysis, it was observed that increased serum resistin levels (odds ratio (OR) = 1.275, 95% confidence interval (CI): 1.065–1.527, p = 0.008), having DM (OR = 4.906, 95% CI: 1.418–16.970, p = 0.012), older age (OR = 1.089, 95% CI: 1.005–1.180, p = 0.036) and elevated hs-CRP (OR = 1.226, 95% CI: 1.010–1.486, p = 0.039) were independent predictors of aortic stiffness in patients with CAD (Table 4).

**Table 4:** Multivariate logistic regression analysis of the factors correlated with aortic stiffness among the 104 patients with coronary artery disease.

Variables	Odds ratio	95% confidence interval	p value
Resistin (ng/ml)(each increase of 1 ng/ml)	1.275	1.065−1.527	0.008*
Diabetes(each has diabetes)	4.906	1.418−16.970	0.012*
Age (years) (each increase of 1 year)	1.089	1.005−1.180	0.036*
high-sensitivity C-reactive protein (mg/dl)(each increase of 0.1 mg/dl)	1.226	1.010−1.486	0.039*

Analysis of data was done using the multivariate logistic regression analysis (adapted factors were diabetes, age, waist circumference, systolic blood pressure, pulse pressure, estimated glomerular filtration rate, high-sensitivity C-reactive protein and resistin).

*p < 0.05 was considered statistically significant.

## Discussion

The results of this study showed that patients with CAD with increased prevalence of diabetes, of older age and having higher waist circumference, SBP, pulse pressure, hs-CRP, iPTH and resistin levels but lower eGFR had high aortic stiffness. Serum resistin together with DM, older age and hs-CRP is an independent predictor for the development of aortic stiffness in these subjects. Moreover, in patients with CAD, DM, older age and higher waist circumference were independent predictors of resistin values.

Arterial stiffening reflects the degenerative changes of extracellular matrix in the media layer and is characterised by elastin fatigue and fracture and collagen deposition that induces loss of arterial elasticity and increased stiffness [18]. In physiological conditions, conduit arteries distend to accommodate large pressure ejections from the heart during systole to facilitate perfusion to tissues during diastole [19]. However, in case of aortic stiffness, the speed of propagation of the arterial pulse through the aorta is increased, and the increased speed of the forward travelling wave implies an earlier reflection of backward travelling wave from the periphery [18]. The backward wave arriving to the ascending aorta during systole instead of during diastole leads to an augmentation of SBP and further increases left ventricular after load and oxygen demand [18, 19]. Reduced aortic elastic recoil and reservoir capacity leads to a fall in DBP, which results in the widened pulse pressure [2]. A lower DBP further reduces coronary artery perfusion and promotes sub-endocardial ischaemia, which is exacerbated by left ventricular hypertrophy [18]. Aging of the arterial system is accompanied by endothelial dysfunction, thickening of the vascular wall and increased stiffening [19]. Our study showed that age, SBP and pulse pressure are higher in patients with aortic stiffness.

Diabetes, metabolic syndrome and chronic inflammation are another pathological state involved in arterial stiffening [18]. Brunner et al. showed that waist circumference was a predictor of aortic stiffening by measuring cfPWV on two occasions 4 years apart in the presence and absence of co-occurring metabolic risk factors and inflammation [20]. Wijkman et al. reported that per each increment of cfPWV by 1 m/s, the hazard ratio of increased CV events increased by 1.142 in patients with type 2 diabetes after adjusting for confounders [21]. A recent study also reported that the duration of diabetes was independently associated with cfPWV in patients with type 2 diabetes [22]. hs-CRP levels were found to be positively associated with cfPWV in 15,302 apparently healthy adults undergoing a general health examination in China [23]. Recent data have defined an important role of inflammation on vascular dysfunction. In vessels, T cell-derived interleukin (IL)-17A acts on smooth muscle cells and adventitial fibroblasts to increase endothelial nitric oxide synthase (eNOS) phosphorylation, reactive oxygen species (ROS) production, collagen synthesis, and chemokine production, leading to a decrease in bioavailable nitric oxide (NO) and impaired vasodilation and increased vascular stiffness [24]. CD4+ CD28− T cells produce high amounts of γ-interferon and tumor necrosis-α (TNF-α) hence becoming proinflammatory cells. Tuttolomondo et al. reported higher peripheral frequency of CD4+ CD28− T cells in acute ischemic stroke compared to control subjects without acute ischemic stroke and higher peripheral frequency of CD4+ CD28− T cells in cardioembolic stroke subtype than other subtypes of ischemic stroke [25]. This group also observed a negative association between reactive hyperemia index value and large artery atherosclerosis subtype and a negative association of cfPWV with cardioembolic subtype [26]. Similar to these studies, our study revealed that patients with CAD in the high aortic stiffness group had higher percentages of DM, waist circumference and hs-CRP levels.

Arterial stiffening with increased blood pressure down induced renal ischaemia and loss of renal autoregulation, which plays an important role in the decreased GFR [27]. Aortic stiffness induced an increase in aortic flow reversal and impedance mismatch reduces antegrade flow into the kidney, which thereby deteriorates renal function in hypertensive patients [28]. Aortic stiffening is independently associated with the rate of change in renal function in patients with chronic kidney disease stages 3 and 4 [29]. Furthermore, increased aortic stiffness is an independent predictor of mortality in patients with stages 2–5 chronic kidney disease [30]. Our results also showed that patients with CAD with high aortic stiffness had lower eGFR than that in those with low aortic stiffness.

Resistin impairs glucose tolerance and insulin action and inhibits adipogenesis in murine 3T3-L1 cells and has been proposed as an adipocyte-secreted factor that is believed to link obesity and type 2 DM [6, 7]. Resistin stimulates endothelin-1 secretion by endothelial cells [31] and increases the expression of endothelial cell adhesion molecules [32]. Serum resistin was found to be associated with an increased risk of both all-cause and CV mortality [33]. Human resistin is predominantly expressed in peripheral blood mononuclear cells and has a role in pro-inflammatory processes [34]. Resistin levels were also found to be associated with plasma CRP levels in the Study of Inherited Risk of Coronary Atherosclerosis (SIRCA) [35]. Plasma resistin levels were found to be inversely associated with eGFR in patients with chronic kidney disease [36, 37]. Our results showed that age, waist circumference, log-Cre and log-hs-CRP positively correlated while eGFR negatively correlated with serum resistin levels in patients with CAD. The multivariate forward stepwise linear regression analysis revealed that DM, age and waist circumference were independent predictors of resistin values in patients with CAD. Windham et al. reported that serum resistin independently correlated with cfPWV in a cross-sectional analysis of data from the Baltimore Longitudinal Study of Aging (BLSA) [38]. Norman et al. also showed that plasma resistin is independently associated with aortic stiffness after adjusting for BP, insulin resistance and general inflammation [9]. Our results also show that serum resistin levels are higher in the high aortic stiffness group. Serum resistin level was also an independent clinical predictor of aortic stiffness in patients with CAD after multivariate analysis.

The limitation of this study is that this was a cross-sectional study with a limited number of patients with CAD and hence the possibility of bias could not be excluded. Another limitation is that pharmacological interventions have been shown to influence cfPWV values or resistin levels in humans. Anti-hypertension β-blocker drugs such as nebivolol and bisoprolol had PWV-lowering effect [39, 40]. Plasma resistin levels were reduced from baseline with barnidipine + losartan treatment but were not reduced with telmisartan + hydrochlorothiazide treatment in patients with hypertension and type 2 DM [41]. Another meta-analysis study did not find any significant change in plasma resistin concentrations following statin therapy [42]. Our results did not show a relationship between anti-hypertension drugs, statins or fibrates and serum resistin levels in the patients analysed in this study. Further studies are required to elucidate the relationship between medication use and resistin levels in patients with CAD.

In conclusion, this study showed that resistin levels were higher in patients with CAD with high aortic stiffness than in those with low aortic stiffness, and together with DM, older age and hs-CRP, resistin level was as an independent predictor for the development of aortic stiffness in these patients. In addition, DM, age and waist circumference positively correlated with resistin levels in patients with CAD.

## Supporting information

S1 Dataset Dataset in the current study. (XLS)
